# Prediction Formula for Pathological Depth of Invasion From Clinical Depth of Invasion in Tongue Squamous Cell Carcinoma (SCC) Stage I/II Cases

**DOI:** 10.7759/cureus.34516

**Published:** 2023-02-01

**Authors:** Mei Hamada, Yasuhiro Ebihara, Saori Yoshida, Naoko Saito, Yuichro Enoki, Hitoshi Inoue, Satoko Matsumura, Mitsuhiko Nakahira, Masanori Yasuda, Masashi Sugasawa

**Affiliations:** 1 Pathology, Saitama Medical University International Medical Center, Saitama, JPN; 2 Pathology, Saitama Medical University, Saitama, JPN; 3 Head and Neck Surgery and Otolaryngology, Saitama Medical University International Medical Center, Saitama, JPN; 4 Preliminary Examination Room, Okayama University Hospital, Okayama, JPN; 5 Radiology, Juntendo University School of Medicine, Tokyo, JPN; 6 Radiology, Saitama Medical University International Medical Center, Saitama, JPN

**Keywords:** lymph node metastasis, formalin fixation, prognostic factor, tongue squamous cell carcinoma, depth of invasion

## Abstract

Background: The depth of invasion (DOI) of tongue squamous cell carcinoma (SCC) is an important prognostic factor. The definition is clear for pathological DOI (pDOI), but the treatment strategy is determined by the preoperative clinical DOI (cDOI). Few studies have investigated the difference between these DOIs. The purpose of this study was to obtain the correlation equation between cDOI and pDOI for Stage I/II tongue SCC and to consider the points to be noted in actual clinical practice.

Methods: In this retrospective study, 58 patients with clinical stage I/II tongue SCC were included. Correlations between cDOI and pDOI were obtained for all 58 cases, as well as for 39 cases which excluded superficial and exophytic lesions.

Results: The overall cDOI and pDOI median values were 8.0 and 5.5 mm, respectively; the 2.5 mm reduction was significant (p < 0.01). The correlation equation was pDOI = 0.81 × cDOI-0.23 (r = 0.73). Furthermore, re-analysis of the 39 cases revealed that pDOI = 0.84 × cDOI-0.37 (r = 0.62). Hence, a derived equation pDOI = 0.84 × (cDOI-0.44) was obtained to predict pDOI from cDOI.

Conclusions: This study indicated that it is necessary to consider contraction due to specimen fixation by subtracting the thickness of the mucosal epithelium. Clinical T1 cases with a cDOI of 5 mm or less had a pDOI of 4 mm or less, and it would be expected to have low positive rate of neck lymph node metastasis.

## Introduction

Oral cancer is the eighth most deadly type of cancer in the world, with squamous cell carcinoma (SCC) of the tongue predominating [1]. The International Consortium for Outcome Research (ICOR) in head and neck cancer reported the importance of the depth of invasion (DOI) in addition to the length of the primary tumor as a prognostic factor [2], and DOI was added as a factor in the new T classification of oral cancer [3-4]. DOI is also associated with lymph node metastasis, and NCCN guidelines in oncology recommend elective neck dissection (END) for stage I/II cases of tongue cancer with a DOI > 4 mm [5]. However, the optimum DOI cut-off value for END varies depending on the report [6-8], and it is reported that it is difficult to define a clear cut-off value [9]. In addition, although the DOI used in these reports is the postoperative pathologically derived DOI (pathological DOI: pDOI), the actual treatment determinant is the preoperative clinically measured DOI (clinical DOI: cDOI). Based on the cDOI, a clinical T classification (clinical T) can be obtained. Therefore, it is important to be aware of the potential difference between cDOI and pDOI and which DOI to use when discussing and making treatment decisions. Previous studies have analyzed the difference between the two DOIs; however, the verification of the correlation between the two is insufficient. The aims of this study were to investigate the correlation between preoperative cDOI and postoperative pDOI to obtain an estimation formula for Stage I/II early tongue cancer, to consider the origin of the formula, and to consider the points to be used for clinical judgment.

## Materials and methods

We investigated clinical stage I/II tongue SCC patients who underwent the initial treatment of primary tongue resection at the International Medical Center of Saitama Medical University from 2007 to 2015. The retrospective protocol was approved by the Institutional Review Board of Saitama Medical University International Medical Center in Japan (Approval No. 18-037), and all methods were performed in accordance with the 1975 Declaration of Helsinki.

**Figure 1:**
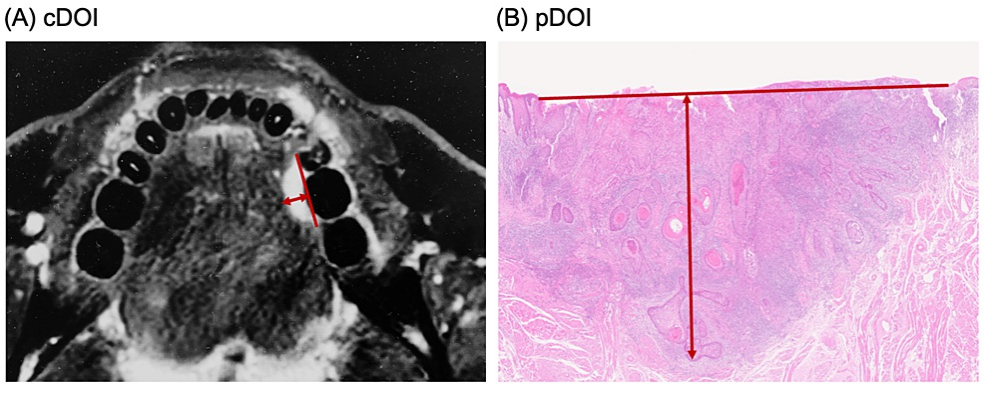
Measurement of depth of invasion. Each image shows the measured cDOI and pDOI, respectively (two-directional arrow). The virtual reference line is drawn by connecting the adjacent mucosal basal portions of the tumor (solid line). (A) cDOI. (B) pDOI. cDOI, clinical depth of invasion; pDOI, pathological depth of invasion

 In this observational study, we collected clinical information from medical records and imaging data and reviewed histopathological specimens. Since MRI is superior in tissue resolution with high reliability [10-11], and reproducibility, cDOI was measured by MRI. MRI measurement of cDOI was performed by drawing a perpendicular line from the reference line (virtual mucosal surface) that connects the tumor and the mucous membrane boundary with a straight line to the deepest part of the tumor (MRI T2 or contrast T1) (Figure 1A) [12-13]. A specialized clinician (EY) measured the data which was confirmed by a radiologist (SN). The minimum value for superficial lesions was evaluated as 1 mm.

 All patients underwent partial resection of the primary lesion. The excised specimen was pin-fixed to a rubber plate, fixed with 10% neutral buffered formalin, sliced every 5 mm, and examined by hematoxylin-eosin-staining. Two oral pathologists (HM and YS) evaluated the maximum thickness of the entire tumor as the pathological thickness (p-thickness), and the perpendicular line drawn from the virtual line connecting the adjacent mucosal basal portions of the tumor to the deepest part of the tumor as the pDOI (Figure 1B) [3-4]. When it was found to be pTis, pDOI was set to 0 mm. Based on the values of p-thickness and pDOI, lesions with a p-thickness > pDOI were defined as exophytic lesions. In addition, we defined a superficial lesion as a case with a DOI of up to 4 mm, which does not require END according to the guidelines. All data were statistically analyzed using R (Easy R) version 11. A significance test was performed with p < 0.05. The Kaplan-Meier method was used for the survival rate; Fisher's exact test was used for the significance test of the contingency table; the relationship between the cDOI and pDOI was analyzed using the Wilcoxon signed rank sum test and linear regression method; and the Pearson correlation coefficient was calculated to quantify the strength of the correlation between the two measurements.

## Results

**Figure 2:**
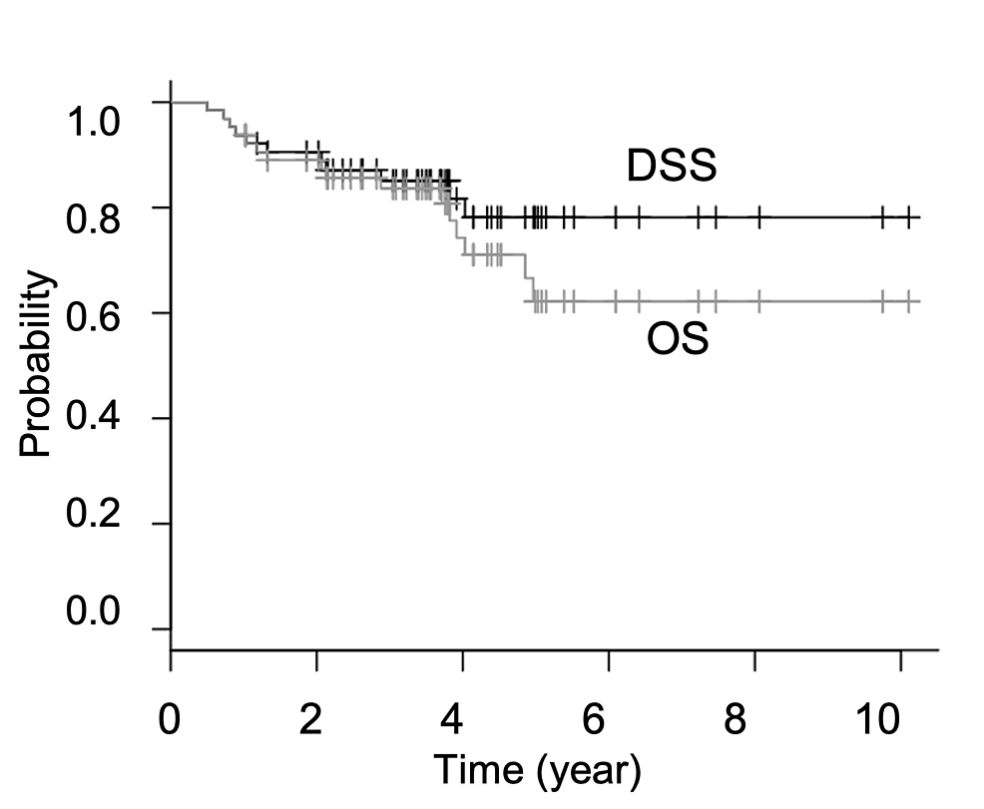
Overall survival and disease specific survival. DSS, disease specific survival; OS, overall survival

Sixty-four cases were collected and six cases were excluded because the primary lesions could not be evaluated by MRI due to artifacts. Therefore, 58 patients who met the criteria were included in the analysis. Clinicopathological features (Table 1) were as follows: age 22-89 years (median 63 years), 43 males (77%), 15 females (23%); clinical T1: T2 = 14 (24%): 44 (76%). The follow-up period was 6-121 months (median 44 months). The final outcomes were 43 survivors (74%); and 15 deaths (26%) of which 10 deaths are due to the primary disease and five deaths due to other causes without tongue cancer. The three-year overall survival (OS) was 83% and the disease specific survival (DSS) was 85%; the five-year OS and DSS were 62% and 78%, respectively (Figure 2).

**Table 1:** Case distribution. cT, clinical T; cDOI, clinical depth of invasion; pDOI, pathological depth of invasion

Variables	ALL (n = 58)
Median age, median years (range)	63 (22-89)
Gender (male vs female)	43 (77%) vs 15 (23%)
cT1 vs cT2	14 (24%) vs 44 (76%)
cDOI, median value (range)	8.0 mm (1-20 mm)
pDOI, median value (range)	5.5 mm (0-25 mm)
Secondary neck lymph node metastasis (+) vs (-)	20 (34%) vs 38 (66%)
Follow up interval (median)	6-121 months (44)
All deaths vs survival	15 (26%) vs 43 (74%)
Disease specific death vs survival	10 (17%) vs 48 (83%)

**Figure 3:**
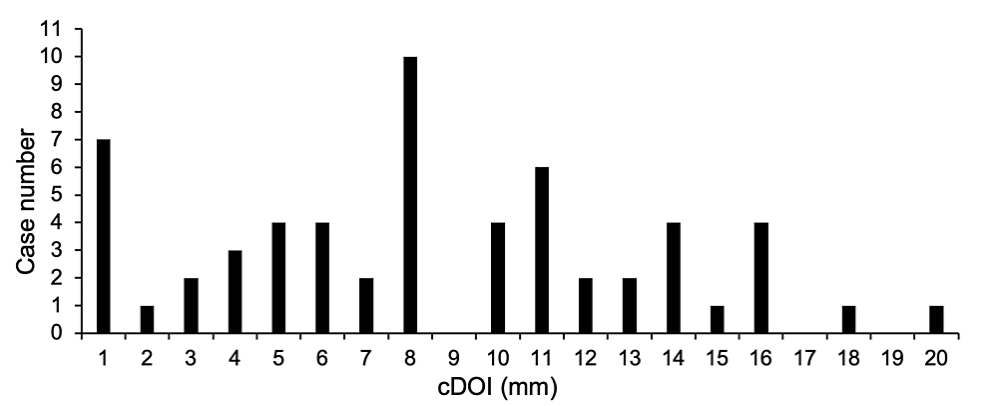
Histogram representations of cDOI. The bar graph shows the distribution of the number of cases by cDOI. cDOI, clinical depth of invasion

**Figure 4:**
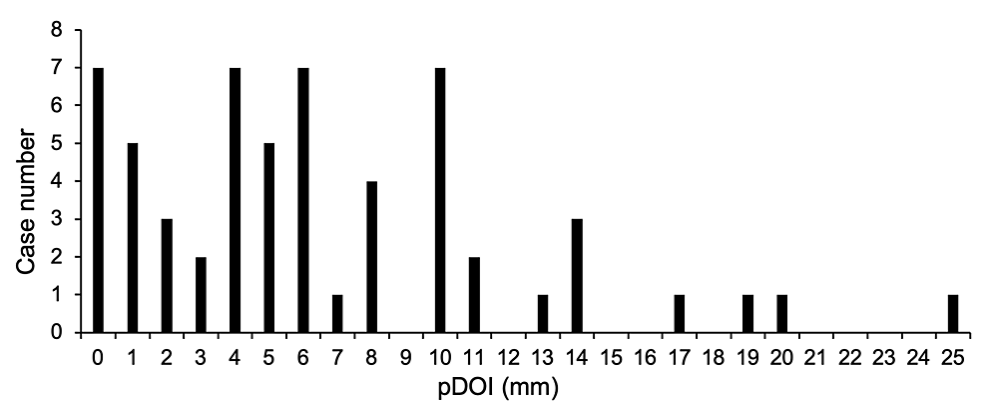
Histogram representations of pDOI. The bar graph shows the distribution of the number of cases by pDOI. pDOI, pathological depth of invasion

 Neck lymph node metastasis occurred in 20 cases (34%), but only one case (cDOI 4.0 mm, pDOI 4.5 mm) out of 13 cases had a cDOI ≤ 4 mm, which was a significantly lower rate (8%) (p = 0.02) [A) in Table 2]. Moreover, there was only one case of neck lymph node metastasis among 17 cases of cDOI ≤ 5 mm, and this rate was significantly low (p < 0.01) [B) in Table 2]. The cDOI and pDOI distributions (histograms) are shown in Figures 3-4. The overall cDOI and pDOI values are as follows; median: cDOI 8.0 mm, pDOI 5.5 mm; mean: cDOI 8.0 mm, pDOI 5.0 mm; range: cDOI: 1-20 mm, pDOI: 0-25 mm. In 17 cases within a cDOI of 5 mm, only two cases were above pDOI 4 mm (12%). The distribution of cDOI was almost uniform, except for prominence in 1 and 8 mm. The pDOI distributions showed a shift to the left compared to the cDOI. The two values showed a significant difference when the Wilcoxon signed rank sum test was used (p < 0.01), showing a median reduction of 2.5 mm when cDOI and pDOI were compared.

**Table 2:** Relation between cDOI and pathological lymph node metastasis and pDOI range. cDOI, clinical depth of invasion; pDOI, pathological depth of invasion

A) Cut-off cDOI 4 mm	pN (-)	pN (+)	Total	pDOI range (mm)
cDOI ≤ 4 mm	12	1	13	0-4.5
cDOI > 4 mm	26	19	45	0-25
Total	38	20	58	(p = 0.02)
B) Cut-off cDOI 5 mm				
cDOI ≤ 5 mm	16	1	17	0-5
cDOI > 5 mm	22	19	41	0-25
Total	38	20	58	(p < 0.01)

**Figure 5:**
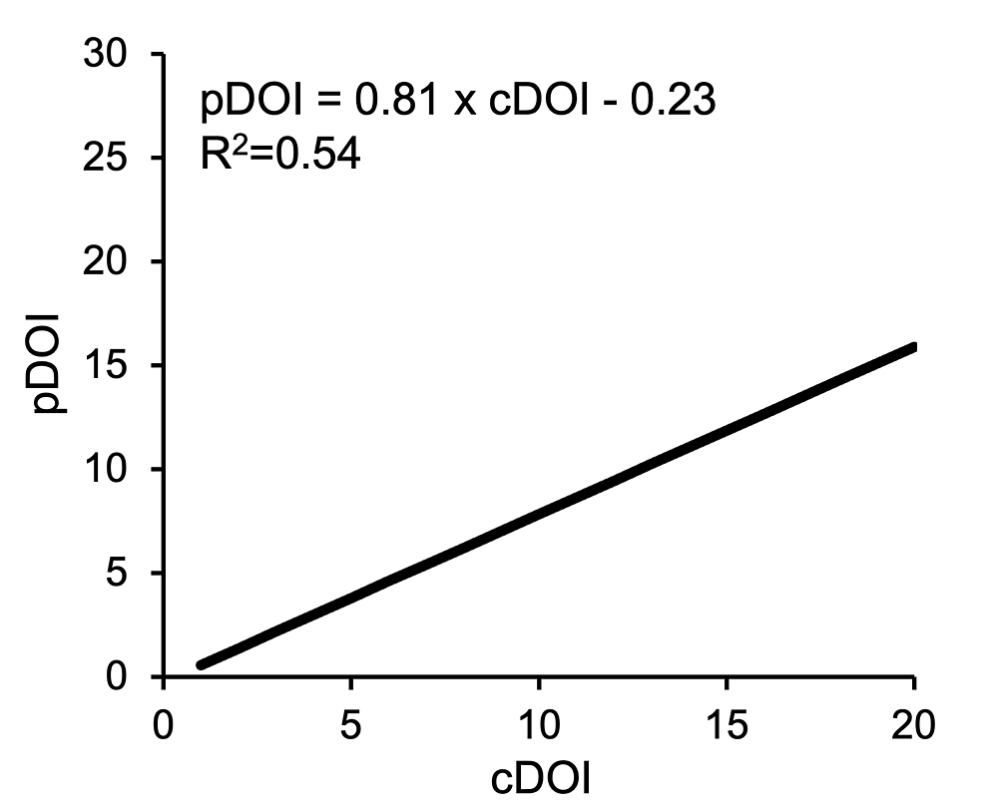
Linear regression equations of cDOI and pDOI (all cases). cDOI, clinical depth of invasion; pDOI, pathological depth of invasion

**Figure 6:**
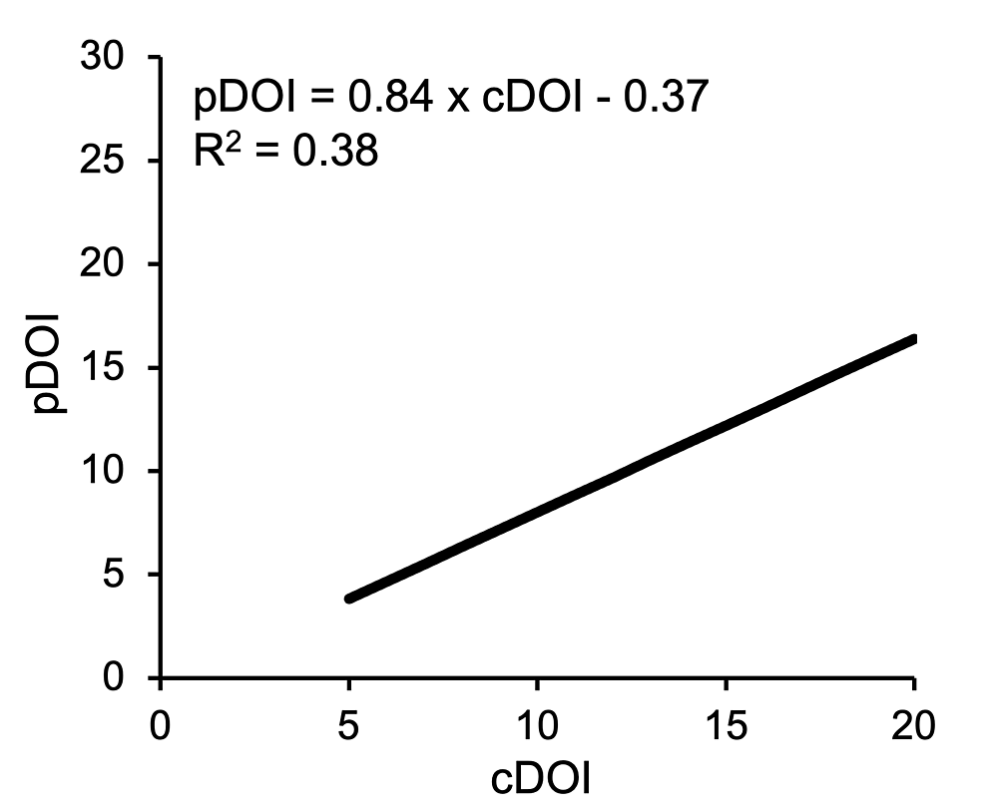
Linear regression equations of cDOI and pDOI (39 cases). cDOI, clinical depth of invasion; pDOI, pathological depth of invasion

Next, the cDOI and pDOI values were plotted and a regression line was drawn using linear regression analysis (Figure 5). Pearson’s correlation coefficient showed a strong correlation (r = 0.73), and the correlation equation was pDOI = 0.81 × cDOI - 0.23. This equation was analyzed by all cases including superficial and exophytic lesions. Therefore, we re-analyzed the data excluding 17 cases, which consisted of 13 cases with a superficial cDOI of less than 5 mm, and four cases of elevated lesions with a thickness > DOI. The result with the 39 cases was pDOI = 0.84 × cDOI-0.37 (r = 0.62) (Figure 6). By factorizing the latter equation, pDOI = (cDOI-0.44) × 0.84, the equation for predicting pDOI from cDOI was obtained.

## Discussion

 In tongue SCC, there are cases in which the prognosis is poor, even with early stage I/II lesions. DOI is the novel component of the T classification of tongue SCC in the American Joint Committee on Cancer (AJCC) 8th edition manual and is an important prognostic factor associated with neck lymph node metastasis [3]. In clinical practice, the clinician evaluates the cDOI by palpation before treatment and by modalities such as CT, ultrasonography (US), and MRI which are used in order to determine the clinical T and to perform END. Preoperative accurate cDOI evaluation is important for treatment planning. However, the current DOI is defined as the pDOI measurement after modification by formalin fixation and preparation of the specimen. The DOI in tongue cancer is evaluated numerically, and thus differs from other gastrointestinal organs (e.g., esophagus, stomach, colon, etc.) that are based on histological structures such as the muscularis mucosa [3]. For this reason, it becomes necessary to interpret the numerical changes due to tissue fixation and so on. That is, there is a discrepancy in that most reports on DOI are based on pDOI that are found after surgery, even though it is the cDOI that is used for actual treatment planning. Clinicians must recognize and judge the difference between the preoperatively evaluated cDOI and the postoperatively evaluated pDOI, but there have been few reports that specifically examined the correlation between the two using a mathematical formula. In this study, we analyzed the correlation between cDOI and pDOI; cDOI was measured by MRI. MRI was used because it is reported to be excellent in reproducible preoperative DOI evaluation with minimal noise between and within evaluators; it is also performed routinely at our hospital [10-11, 14]. The extent of a tumor, as assessed on MRI images, is reported to correlate with the histological extent of tumor invasion [15-16]. The pathological DOI was evaluated by the method described in the AJCC manual and the fixation method and measurement at our hospital are also general methods.

 As a result, the median value was significantly different between cDOI 8.0 mm and pDOI 5.5 mm (p <0.01), and the difference in the median value was 2.5 mm. In addition, Terada et al. reported a median difference of 2.7 mm for Stage I/II tongue cancer [9] and Murakami et al. reported 2-3 mm for Stage II tongue cancer [11], both of which are considered to be values that cannot be ignored in clinical practice. The prediction formula for all cases is pDOI = 0.81 × cDOI-0.23, and the Pearson correlation coefficient shows a strong correlation (r = 0.73) indicating that the lesion shrinks upon pathological evaluation.

 The common cause to all cases was considered to be excision preparation after resection and the effect of formalin fixation [17-18]. It is generally known that a dimensional change occurs in the process of preparing a pathological tissue specimen. Formalin acts by binding to amino groups, thus stabilizing and coagulating proteins. This reaction can cause cell contraction and distortion. In addition, after fixation with formalin, it is also affected by dehydration with alcohol, compression during embedding in paraffin, and sufficient extension of paraffin section [19-20]. Although there are few reports on the contraction rate due to these effects on the tongue, the contraction rate that we found was close to 23.5%, as reported by Mistry et al. [21].

 The preoperative overestimation of results is due to inflammatory effects such as bleeding and necrosis; misevaluation of exophytic lesions including thickness [16], undetermined microinvasion in the deepest area, and unknown progress during waiting for surgery. In particular, it has been reported that for superficial lesions with a pDOI of less than 5 mm, cDOI and pDOI does not correlate because image evaluation becomes difficult [13]. Therefore, we considered that a better correlation equation could be obtained by the analysis excluding superficial lesions and exophytic lesions [12], and a prediction equation was obtained for the 39 cases. The result was pDOI = (cDOI-0.44) × 0.84. This equation shows that when predicting the pDOI from the cDOI, the thickness of the mucosal epithelium of 0.44 mm must be subtracted and the contraction rate of 16% must be taken into consideration. On the tongue, Lwin et al. reported a coefficient of correlation of 0.87 [22], which was similar to our result. Regarding the thickness of the oral mucosa epithelium, it is almost consistent with this coefficient of 0.44 mm [23]. Predicting a pDOI from a cDOI using this formula, preoperative cDOI of 5 mm becomes pDOI of 4 mm. This means that the T1 maximum DOI value of 5 mm measured preoperatively corresponds to the DOI threshold of 4 mm which is the END recommendation of the NCCN guideline (dependent on pathological data). In 17 cases with a cDOI of up to 5 mm evaluated preoperatively by MRI in this study, lymph node metastasis was extremely low at one case (6%), and the benefit of END was poor. Fifteen patients remained within a pDOI of 4 mm, and there was no increase in the prediction of lymph node metastasis due to the cut-off difference between the cDOI of 4 and 5 mm. Therefore, for clinical T1 lesions with a cDOI of 5 mm or less (as measured by preoperative MRI), pDOI is expected to be within 4 mm. Additionally, even if the wait-and-watch policy is selected as the treatment instead of END, the risk of lymph node metastasis does not increase, and it does not contradict the NCCN guidelines. Larson also concluded that the risk of metastases within 4 mm of pDOI is extremely low [24].

 Similarly, a preoperative cDOI of 10 mm is expected to be a pDOI of 8 mm. Many reports have used a pDOI of 8 mm as a cut-off value for lymph node metastasis and survival rate [12, 25-27], corresponding to the boundary value cDOI 10 mm of clinical T2 and T3, which is in line with clinical judgment, useful for risk prediction in actual clinical practice. In the future, for more accurate prediction of lymph node metastasis, it is necessary to examine cases with different cDOI and pDOI and to consider comprehensively together with PET/CT, US, MRI, and other modalities.

## Conclusions

The pDOI estimation formula from cDOI using MRI is pDOI = (cDOI-0.44) x 0.84, and it is necessary to subtract the thickness of the mucosal epithelium and consider contraction due to specimen fixation when predicting the pDOI from the cDOI. Clinical T1 cases, evaluated as a cDOI of 5 mm or less by MRI, would result in a pDOI of less 4 mm and be exposed to low positive rate of neck lymph node metastasis.
